# Use of EP3533-Enhanced Magnetic Resonance Imaging as a Measure of Disease Progression in Skeletal Muscle of *mdx* Mice

**DOI:** 10.3389/fneur.2021.636719

**Published:** 2021-06-17

**Authors:** Alexander Peter Murphy, Elizabeth Greally, Dara O'Hogain, Andrew Blamire, Peter Caravan, Volker Straub

**Affiliations:** ^1^The Institute of Cancer and Genomics, Birmingham University, Birmingham, United Kingdom; ^2^The John Walton Muscular Dystrophy Research Centre, Institute of Translational and Clinical Research, Newcastle University, Newcastle upon Tyne, United Kingdom; ^3^Newcastle Magnetic Resonance Centre, Translational and Clinical Research Institute, Newcastle University, Newcastle upon Tyne, United Kingdom; ^4^Department of Radiology, Martinos Center for Biomedical Imaging, Harvard Medical School, Massachusetts General Hospital, Charlestown, MA, United States; ^5^Newcastle upon Tyne Hospitals NHS Foundation Trust, Newcastle University, Newcastle upon Tyne, United Kingdom

**Keywords:** EP3533, muscular dystrophy, fibrosis, magnetic resonance image, *mdx* mouse model

## Abstract

As putative treatments are developed for Duchenne muscular dystrophy (DMD), sensitive, non-invasive measures are increasingly important to quantify disease progression. Fibrosis is one of the histological hallmarks of muscular dystrophy and has been directly linked to prognosis. EP3533 is a novel contrast agent with an affinity to collagen 1 that has demonstrated a significant and high correlation to *ex vivo* fibrosis quantification. Halofuginone is an established anti-fibrotic compound shown to reduce collagen skeletal muscle fibrosis in murine models of DMD. This experiment explored whether EP3533 could be used to detect signal change in skeletal muscle of *mdx* mice before and after a 12 week course of halofuginone compared to controls. Four age-matched groups of treated and untreated mice were evaluated: 2 groups of *mdx* (*n* = 8 and *n* = 13, respectively), and 2 groups of BL10 mice (*n* = 5 and *n* = 3, respectively). Treated mice received an intraperitoneal injection with halofuginone three times per week for 12 weeks, with the remaining mice being given vehicle. Both *mdx* groups and the untreated BL10 were scanned at baseline, then all groups were scanned on week 13. All subjects were scanned using a 7T Varian scanner before and after administration of EP3533 using a T1 mapping technique. Mice underwent grip testing in week 13 prior to dissection. Skeletal muscle was used for Masson's trichrome quantification, hydroxyproline assay, and immunofluorescent antibody staining. Untreated *mdx* mice demonstrated a significant increase in R1 signal from pre- to post-treatment scan in three out of four muscles (gastrocnemius *p* = 0.04, hamstrings *p* = 0.009, and tibialis anterior *p* = 0.01), which was not seen in either the treated *mdx* or the BL10 groups. Histological quantification of fibrosis also demonstrated significantly higher levels in the untreated *mdx* mice with significant correlation seen between histology and EP3533 signal change. Forelimb weight adjusted-grip strength was significantly lower in the untreated *mdx* group, compared to the treated group. EP3533 can be used over time as an outcome measure to quantify treatment effect of an established anti-fibrotic drug. Further studies are needed to evaluate the use of this contrast agent in humans.

## Introduction

Duchenne muscular dystrophy (DMD) is caused by mutations in the *DMD* gene and is inherited in an X-linked recessive fashion. DMD is considered the most commonly inherited muscle disease in childhood, with an incidence of 1 in 3,500–6,000 live male births (1–3). Dystrophin, the protein product of the *DMD* gene, is a sub-sarcolemmal cytoskeletal protein present in all muscle (4). The phenotype of patients with DMD is of progressive weakness of the skeletal, respiratory and cardiac muscles. DMD is considered a devastating, life-limiting condition (3, 5).

Dystrophin-deficient muscle results in a loss of structural membrane integrity of muscle fibres during cycles of contraction and relaxation. The damaged sarcolemma leads to an influx of cations such as calcium and sodium (6) into the intracellular compartment and consecutively to myofiber necrosis and chronic inflammation. Subsequently there is an accumulation of fibrosis and adipose tissue within the muscle (7). Higher degrees of endomysial fibrosis in skeletal muscle has been associated with a worse prognosis in neuromuscular disease (8). Sensitive and preferably minimally invasive techniques are increasingly important to evaluate putative therapies by quantifying fibrosis *in vivo* as a biomarker of disease progression.

EP3533 is a gadolinium-based MRI contrast agent which has an affinity for type 1 collagen (9). EP3533 has been successful in quantifying and staging fibrosis in several murine models (9–13) as well as quantifying anti-fibrotic therapy response (14). EP3533-enhanced MRI has been previously evaluated in a murine model of DMD, quantifying fibrosis in skeletal and cardiac tissues (15). There was a significant linear correlation demonstrated between *ex vivo* measures of fibrosis and skeletal muscle measurement of R1 change using EP3533-enhanced MRI (gastrocnemius: *r* = 0.83, *P* = 0.001; tibialis anterior: *r* = 0.73, *P* = 0.01) (15). While there is evidence that EP3533-enhanced MRI could be used to quantify fibrosis, it is unclear whether it is sensitive enough to detect disease progression in *mdx* mice and able to demonstrate changes in response to treatment as an outcome measure.

Halofuginone is a potent anti-fibrotic which inhibits phosphorylation of Smad 3 and attenuates gene expression of collagen-α1 in fibroblasts. These effects have been shown to lead to a reduction in collagen deposition and fibrosis (16, 17). Halofuginone has been trialled as an anti-fibrotic in the treatment of *mdx* mice with success *in vivo* (17–19). Studies have shown that halofuginone administration to young and old *mdx* mice have demonstrated significant improvements in measures of fibrosis and skeletal muscle function (18, 19).

The primary aim of this study was to determine whether EP3533-enhanced quantitative MRI could be used to demonstrate significant differences between *mdx* mice treated with an established anti-fibrotic (halofuginone). The secondary aim was to evaluate whether EP3533-enhanced quantitative MRI correlated significantly to *ex vivo* measures of fibrosis and a measurement of muscle function.

## Materials and Methods

Four groups of age-matched (16 ± 3 weeks) *mdx* (C57BL/10ScSn-mdx/J, Jackson, Maine USA)and BL10 (C57BL/10ScSnOlaHsd, wild type, WT, Harlan Laboratories, Indianapolis, USA) male mice were utilised for the study. Groups were split to receive either vehicle (150 μl of 5% dimethyl sulfoxide and 0.9% saline) or halofuginone (7.5 μg) dissolved in the same solution (18, 20). All groups received vehicle or halofuginone via intraperitoneal injections three times per week for 12 weeks. The treated *mdx* group (*n* = 8), untreated *mdx* group (*n* = 13) and the treated BL10 mice (*n* = 3) would undergo baseline MRI scans with EP3533. The untreated BL10 group (*n* = 5) would be scanned at follow up (week 13) only. All groups underwent grip strength testing at week 13, then scanning of the lower limbs. Following this, mice were humanely killed and dissected for *ex vivo* quantification of fibrosis.

### MRI Protocol

Animals were anaesthetised using 5% isoflurane in 0.5 L/min of oxygen, once induced, anaesthesia was maintained at 1 to 2% isoflurane. Hair removal cream was used to clear two patches of skin for contact with ECG electrodes, the tail vein cannulated and a line containing the EP3533 attached. The mouse was placed on a sled, incorporating ECG electrodes for cardiac imaging (Dazai, Canada) and positioned in a 39 mm internal diameter birdcage radiofrequency coil (Rapid Biomedical GmbH, Germany). Core body temperature was maintained using a warm air heating system interfaced to a rectal temperature monitoring probe and heart rate and respiration monitored (SA Instruments, NY).

Magnetic resonance scanning was performed on a 7-Tesla micro-imaging system (Varian; Agilent Technologies, Santa Clara, United States) equipped with a 12 cm micro-imaging gradient insert (maximum gradient, 40 mT/m). Following administration of 20 μmol/kg of EP3533, based on a previous study (15), subjects underwent all imaging at 60–70 min post-infusion using T1 weighted gradient recalled echo (GRE) imaging and then a gradient echo multi-slice inversion-recovery Look-Locker (gemsIR-LL). The T1 mapping sequence was performed on both upper and lower portions of the rear legs using a gemsIR-LL with 1 slice of 1 mm thickness selected through lower leg muscles with the following parameters: repetition time (TR) inversion = 5 s, 10 inversion times (from 0.1 to 5 s), TR/echo time (TE) = 9.68/4.86 ms, echo train length (ETL) = 4, flip angle (FA) = 4°, Field Of View (FOV) 30 × 30 mm, matrix = 128 × 128. The T1 weighted GRE acquired pre and post-contrast enhancement was acquired with TR/TE = 9.46/4.75 ms, FA = 20°, FOV = 30 × 30 × 30 mm, matrix 128 × 128 × 128, band width = 20 KHz.

For the lower leg muscles, mice were positioned with the upper border of the field of view beginning at the lower border of the knee. For the upper leg, mice were positioned with the centre of the knee joint as close as possible to the centre of the radiofrequency coil. Four slices were used to obtain the gemsIR-LL, two positioned above (+0.6 mm, 1.2 mm) and two below the knee joint (−0.6 mm, −1.2 mm). This enabled homogeneity in positioning for analysis.

All procedures performed were in accordance with the ethical standards of directive 2010/63/EU of the European parliament and under the auspices of the terms of the animals (scientific procedures) act 1986 and project licence PB3CA650C, authorised by the Home Secretary, Home Office, United Kingdom.

### MRI Analysis and Region of Interest Selection

All scans were analysed using “Aedes software” (21). T1 maps were created by pixel-wise fitting of the lower limb datasets to the standard inversion recovery Look-Locker equation. Lower limb muscle ROIs were interactively defined in tibialis anterior and gastrocnemius muscles, based on previous MRI-based murine studies (22). Application of the ROIs to T1 maps were used to determine the change in R1 values in skeletal and cardiac muscles pre and post-contrast.

### *Ex vivo* Fibrosis Quantification

Mice were humanely killed immediately after the second scan. The tibialis anterior, gastrocnemius, hamstring and quadriceps muscles were harvested. Samples were snap frozen in liquid nitrogen cooled isopentane and stored at −80°C. The left-sided muscles were collected for quantification of fibrosis markers via hydroxyproline, while right-sided muscles were obtained for histological analysis. In all muscles cryosections were cut 8 μm thick. To ensure more coverage of the muscle to detect focal fibrosis, sections were spaced 200 μm apart.

Histological staining was used to quantify fibrosis and confirm disease pathology. Cryosections were stained with Masson's trichrome (MaTr), haematoxylin and eosin (H&E) and immunofluorescent staining for collagen 1.

The percentage of fibrosis was quantified using the MaTr staining and regions of interest (ROIs) analysis via imaging software (Fiji 64 bit). Fibrosis was calculated as a percentage of the total area of the muscle section. A modified hydroxyproline assay was also used to quantify fibrosis in muscle (23).

### Pharmacokinetics Sampling

To confirm serum levels of halofuginone, pharmacokinetics (PK) was performed in four halofuginone-naive *mdx* mice of similar ages to the experimental groups, using liquid chromatography—mass spectrometry/mass spectrometry (LC-MS/MS). Venesection of the saphenous vein was performed using a needle and capillary tubes after administration of halofuginone. To keep within UK Home Office guidance for volume of venesection per mouse, the samples were taken from four mice at two different time points each (24). From each mouse two samples of 150 μl were taken, with the second a terminal sample. PK levels were measured at: pre-injection, 5, 15, and at 30 min, then at 1, 2, 4, 8, and 24 h. These were stored as serum in lithium-heparin bottles and stored at −80°C. LC-MS/MS allowed quantification against a calibration curve from 0.5 to 1000 ng/ml. A stock solution of halofuginone in DMSO (5%) and saline (0.9%) was provided at a concentration of 10 mM as well as serum from a halofuginone naive *mdx* mouse. Quality control samples (3, 30, and 700 ng/mL) and mouse samples were mixed with organic solvent (methanol) containing a mixture of three generic internal standards. Samples were treated at a ratio of 3:1 solvent to sample. Following protein precipitation, samples were then centrifuged (30 min at 5,000 g). The resulting supernatant was diluted with double distilled water at a ratio of 2:1 parts of supernatant in a 96-well-plate. The plate was sealed, vortex mixed and analysed by LC-MS/MS with incurred samples quantified from the calibration line.

### Functional Assessment of Grip Strength

Prior to the final scan, a measure of muscle function was assessed using a grip strength apparatus (BioSeb, Chaville, France), with both two and four limb assessment performed. These were tested using a “T” shaped bar attachment and were placed upon the apparatus before being pulled gently backwards by the tail. Mice had three attempts with at least a 1 min of rest between assessments. To reduce bias the same examiner performed the test, in the same temperature controlled, quiet environment. The weight and the maximal recorded value from the three attempts was used to calculate the normalised force (force/body weight) (25).

### Statistical Analysis

All statistics were calculated using SPSS (version 23). Correlation is reported as a Pearson coefficient. Student's *t*-test was used to compare means, with *p* < 0.05 considered significant. One way anova was applied to compare grip test results with *post-hoc* analysis performed using the Tukey-Kramer test.

## Results

All mice (*mdx n* = 21, BL10 *n* = 6) tolerated halofuginone treatment, vehicle and EP3533 administration with no side effects attributed to the drug and no significant weight difference was seen between treated and untreated groups. There were no significant differences between BL10 mouse groups in terms of weight, functional assessment, *ex vivo* measures of fibrosis or baseline and follow up R1 change. The BL10 groups were therefore considered together for comparison with *mdx* mouse groups.

### Halofuginone Treatment Effects

**Figure 1:**
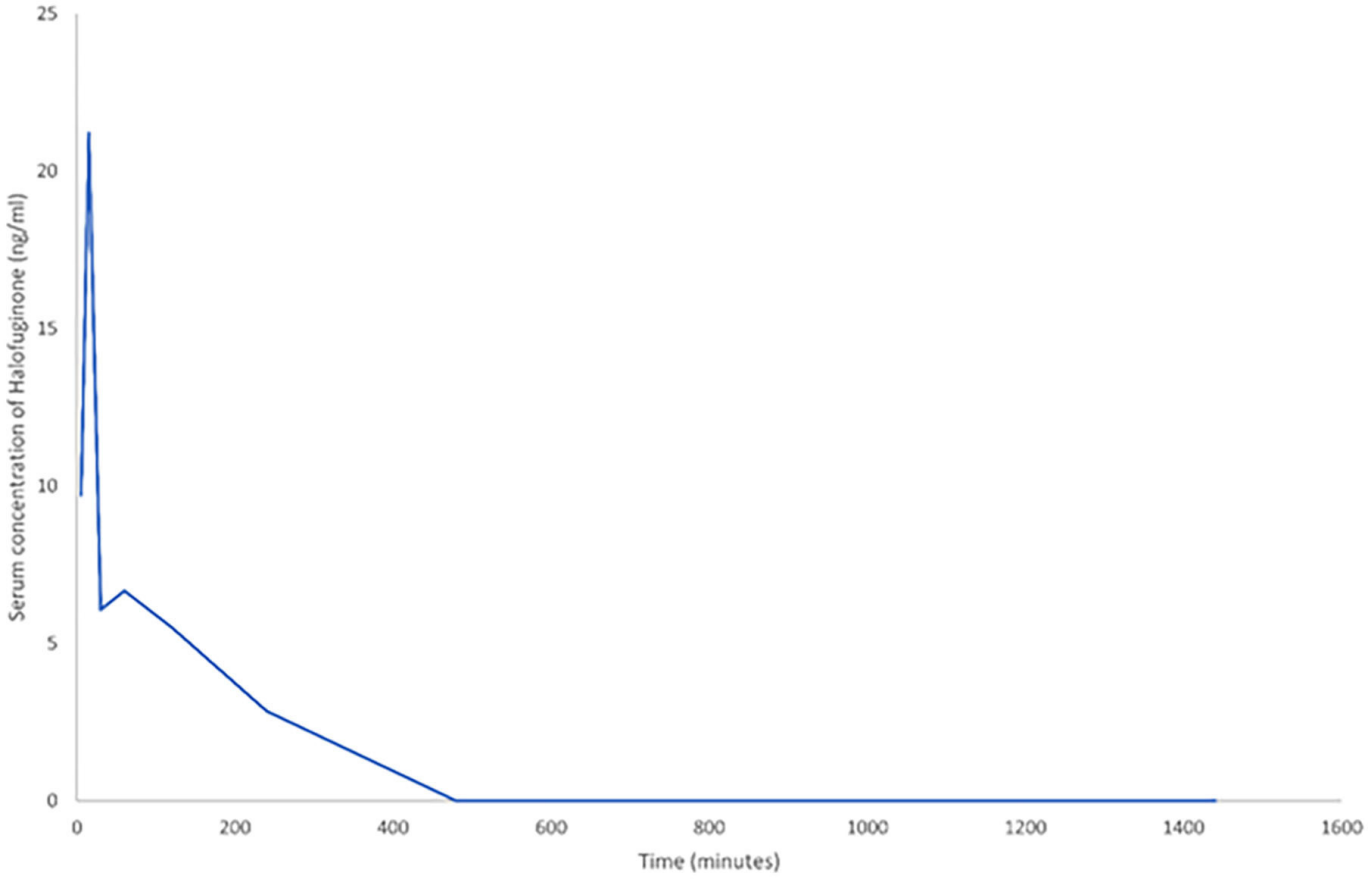
A line graph demonstrating the change in serum halofuginone post-intraperitoneal (IP) administration of 7.5 μg (*n* = 4). To allow inclusion of all time points, time is measured in minutes. The last five data points are at 1, 2, 4, 8, and 24 h respectively.

Pharmacokinetic results demonstrated that halofuginone was detectable in serum at six of the eight time points. The concentration range was from 2.85 to 21.2 ng/ml with the highest peak at 5 min post-administration, these findings suggest successful administration at a concentration expected to induce a treatment effect (Figure 1). Area under the curve analysis was 913.68 ng/ml/min.

### *Ex vivo* Fibrosis Quantification

**Figure 2:**
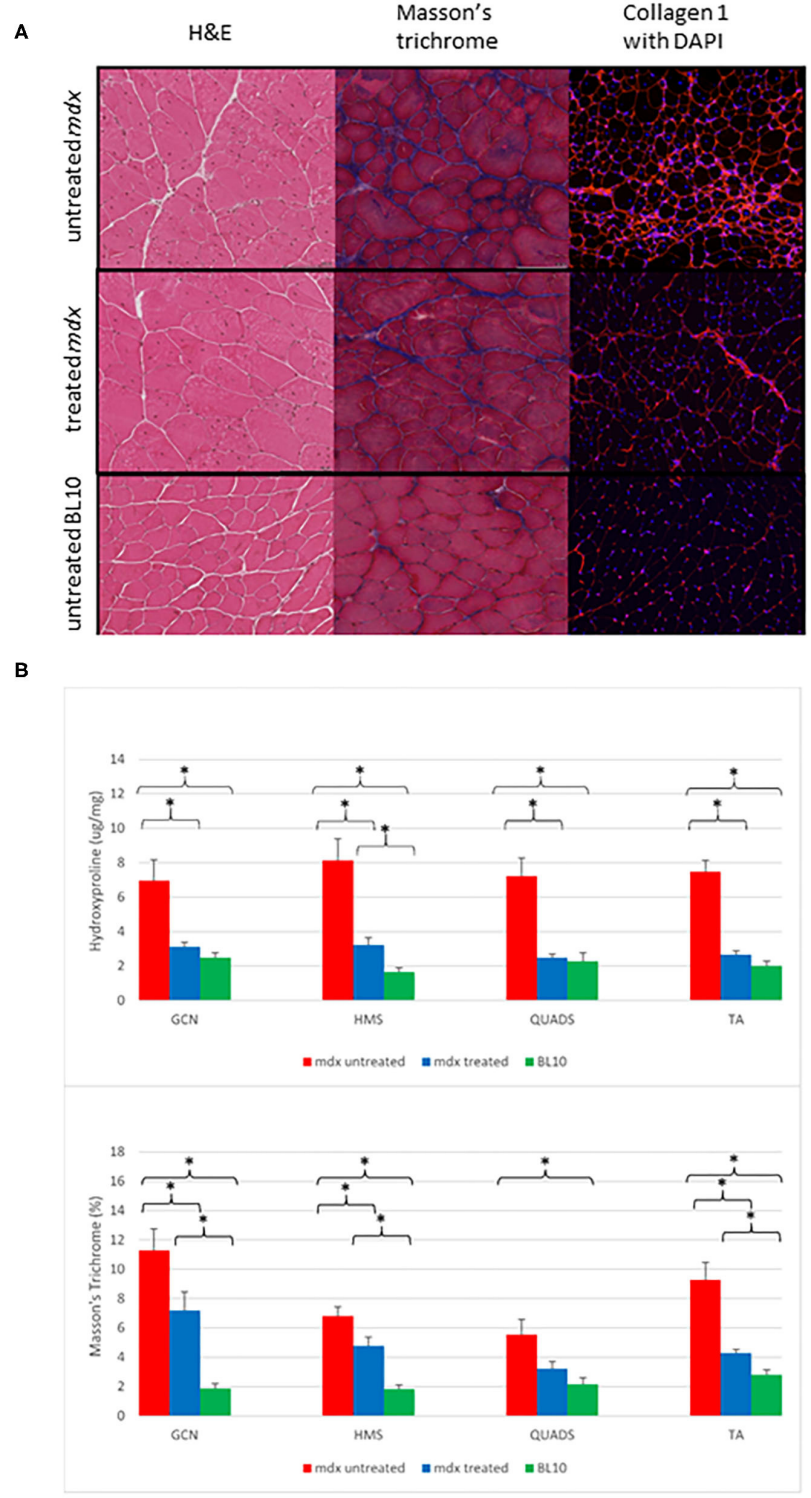
**(A)** Examples of transverse sections from gastrocnemius (GCN) muscles. The left column shows H&E staining at ×20 magnification demonstrate that the *mdx* mouse groups have evidence of more centrally located nuclei, varying myocyte size and areas of fibrosis (white). The middle column demonstrates Masson's trichrome staining in ×20 magnification. Fibrosis is shown as dark blue, the mdx untreated group show diffuse fibrosis throughout with the BL10 group showing very small amounts of fibrosis, the treated mdx mouse group shows fewer areas of blue staining compared to the untreated mdx group. The right column is immunofluorescent staining for collagen 1 with DAPI, areas of red are areas with collagen 1, nuclei are highlighted as blue (Magnification ×10). Similar to the Masson's trichrome staining, the untreated *mdx* mice have large amounts of diffuse fibrosis evident across the whole slide, with the BL10 slide only showing collagen 1 staining in the sarcolemma in an ordered fashion. The treated *mdx* mice demonstrate an amount of collagen staining in between these. **(B)** Bar charts to show the differences in *ex vivo* quantification of fibrosis within muscle between treated *mdx*, untreated *mdx* and BL10 groups. **(A)** hydroxyproline quantification, **(B)** MaTr quantification. Significant differences are seen between the all muscles of the untreated *mdx* mice and the BL10 group using bother measures of fibrosis. Using Masson's trichrome (MaTr) quantification, all muscles had significantly lower amounts of fibrosis in the treated *mdx* group compared to the untreated. Hydroxyproline quantification identified 3/4 of these muscles as significantly lower in the treated mdx group. Error bars represent standard error. *p < 0.05, GCN, Gastrocnemius, HMS, Hamstrings, QUADS, Quadriceps, TA, Tibialis anterior.

**Figure 3:**
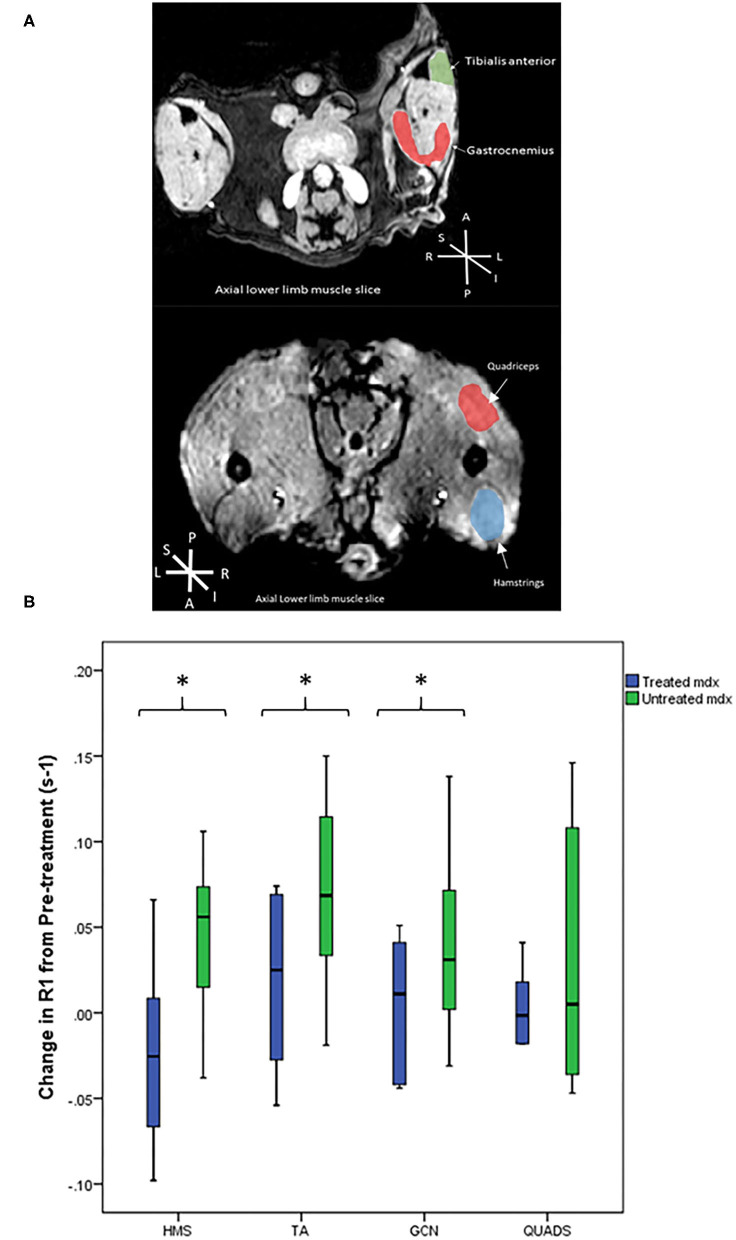
**(A)** Representative T1 weighted axial MRI scans through hind limbs with regions of interest labelled. P, posterior, A, anterior, L, left, R, right, S, superior, I, inferior. **(B)** Box plots to compare the difference in R1 change between the *mdx* groups from pre-treatment to post-treatment scans. The treated *mdx* mice did not show significant increases in R1 post-treatment whereas the untreated *mdx* group showed significant increases in R1 change over time in all three muscles: gastrocnemius *p* = 0.04, hamstrings *p* = 0.009, and tibialis anterior *p* = 0.01. *p < 0.05, GCN, Gastrocnemius, HMS, Hamstrings, QUADS, Quadriceps, TA, Tibialis anterior.

Histological analysis demonstrated the diffuse nature of the fibrotic changes in *mdx* mouse muscle and comparative differences between groups (Figure 2). All treated *mdx* muscles demonstrated significantly lower mean levels of fibrosis compared to untreated *mdx* using hydroxyproline assay. Comparing treated to untreated *mdx*, MaTr quantification was significantly lower in three of the four muscles, with the exception of the QUADS (*p* = 0.06). These results suggest that levels of fibrosis were lower in the majority of muscles in treated *mdx* groups compared to the untreated *mdx* groups (Figure 3).

### Functional Assessment

The mouse groups were compared using a one-way Anova test. Using the two-limb grip strength measure the untreated *mdx* mouse group was significantly weaker than the other groups (*F* = 6.08, *p* < 0.03). *Post-hoc* analysis demonstrated a significant difference between the untreated and the treated *mdx* mice (untreated *mdx* 21.5 mN/g vs. treated *mdx* 37.0 mN/g). There were no statistically significant differences between the BL10 groups and the treated *mdx* mice (*p* = NS).

### R1 Change in EP3533 Enhanced MRI

Pre-treatment scans demonstrated no significant differences between the treated and untreated *mdx* mouse groups. Figure 3A shows representative T1 weighted axial MRI scans through hind limbs with regions of interest labelled. In the pre-treatment scans R1 change of all muscles were significantly higher at 60 min in the combined *mdx* groups than the BL10 at 60 min. These findings suggest that there was no detectable difference between the two *mdx* groups at baseline and that EP3533-enhanced scans were sensitive enough to discern between BL10 and *mdx* groups at an early age (12 weeks).

In the post-treatment scan, there were no significant differences between the untreated and treated BL10 groups in any of the muscles at any of the time points. No significant differences were seen between treated and untreated *mdx* groups in absolute R1 value at the post-treatment scan. When comparing the R1 change over time from pre-treatment to post-treatment scan there was a significant increase in three out of four of the muscle of the untreated *mdx* group (GCN *p* = 0.04, HMS *p* = 0.009, and TA *p* = 0.01; Table 1, Figure 3B). No significant change was seen over time in any of the muscles in the other groups.

**Table 1:** Table to show absolute R1 change at both the pre- and post-treatment scans.

	**Pre-treatment scan change in R1 from baseline (s^**−1**^)**	**Post-treatment scan change in R1 from baseline (s^**−1**^)**	**Correlation of change in R1 at post-treatment scan to Masson's Trichrome^†^**	**Correlation of change in R1 at post-treatment scan to hydroxyproline assay^**†**^**
*mdx* TA	0.11 ± 0.05^*^	0.14 ± 0.06^*^	0.84^**^	0.58^*^
BL10 TA	0.11 ± 0.02	0.07 ± 0.01		
*mdx* GCN	0.10 ± 0.04^*^	0.10 ± 0.05^*^	0.80^**^	0.59^*^
BL10 GCN	0.04 ± 0.05	0.04 ± 0.05		
*mdx* HMS	0.11 ± 0.04^*^	0.12 ± 0.04^*^	0.59^*^	0.41^*^
BL10 HMS	0.03 ± 0.04	0.06 ± 0.06		
*mdx* QUADS	0.09 ± 0.12^*^	0.09 ± 0.05^*^	0.59^*^	0.24
BL10 QUADS	0.001 ± 0.12	0.04 ± 0.06		

*Also demonstrates the high degree of significant Pearson correlation between R1 change at post-treatment scan and ex vivo measures of fibrosis*.

*TA, Tibialis anterior; GCN, Gastrocnemius; HMS, Hamstrings; QUADS, Quadriceps*.

* *P < 0.05*.

** *P < 0.001*.

† *BL10 and mdx considered together*.

### Correlation of EP3533-Enhanced MRI Findings to *ex vivo* Measures of Fibrosis

R1 values in all muscles at the post-treatment scan correlated significantly with Masson's trichrome (Table 1). R1 change at post-treatment correlated linearly in three out of four muscles with hydroxyproline. The strongest correlations to *ex vivo* measures of fibrosis were in the gastrocnemius muscle (Table 1).

### Correlation of EP3533-Enhanced MRI to Functional Assessments

None of the two limb force assessments significantly correlated to the change in R1 values from the post-treatment scan, in either the individual muscles or all muscles grouped together. Considering all muscles together the change in R1 values including the pre and post-treatment scans correlated significantly, albeit weakly (*r* = −0.33, *p* = 0.004). The correlation may suggest that there is an association between R1 change at post treatment and muscle function, the lack of a strong correlation may be due to the other confounding factors that influence muscle function.

## Discussion

Muscular dystrophies such as DMD are slowly progressive conditions, the histological hallmarks include inflammation, fibrosis, and fat replacement of healthy muscle. Eventually, secondary to this pathology, the muscles are unable to function and clinical weakness is evident. Clinical trial outcome measures are biomarkers of disease progression that are increasingly important to test putative therapies. In clinical trials involving neuromuscular disease the most commonly used outcome measures are assessing muscle strength and muscle function. Use of a non-invasive outcome measure based on earlier pathology provides a potentially more sensitive biomarker to disease progression over a shorter period. Such outcome measures are sorely needed as muscular dystrophies may take several years to progress clinically, this makes drug development time consuming and expensive. This is the first study to look at use of EP3533 as an outcome measure in muscular dystrophy. Previous studies have shown that EP3533-enhanced MRI can demonstrate sufficient sensitivity to fibrosis *in vivo* to accurately stage disease, demonstrate disease progression and quantify fibrosis in organs with low proton density (9, 10, 12–14). This study showed that EP3533 was able to demonstrate a treatment effect as well as demonstrating strong correlations with *ex vivo* measures of fibrosis, and a weaker correlation to a functional assessment.

Halofuginone was chosen as an anti-fibrotic as it has previously demonstrated that it can induce a reduction in collagen and an improvement of muscle function in murine models (18, 19). Halofuginone is currently being evaluated in a clinical trial (26). This study further supported the use of halofuginone to reduce fibrosis *in vivo* and to improve muscle function. In this experiment the *mdx* treated and BL10 groups were not significantly different in a number of ways including: absolute values of R1 change over time, functional assessments, and degree of *ex vivo* fibrosis in several muscles (Figure 3). Other anti-fibrotics have been trialled in DMD patients, though in humans factors such as fat replacement may reduce the effectiveness of these drugs (27–29). Anti-fibrotics do have the advantage of not being mutation dependent, unlike a number of recent medications aimed at increasing dystrophin production (30–32). As anti-fibrotic agents are potential drug candidates to treat muscular dystrophies, we tested if EP3533-enhanced MRI could be used as an outcome measure, as it has been shown to be successful at monitoring fibrosis progression over time in murine models. A further advantage of EP3533 is that due to the non-invasive nature of the test fewer animals would need to be sacrificed to demonstrate interim changes in experiments.

Currently the only validated way to quantify fibrosis in human skeletal muscle is via muscle biopsy (33). Muscle biopsy is invasive, risks sampling error and includes the risk of a general anaesthetic in patients with reduced respiratory function. EP3533, by contrast, has been shown to be able to measure change in skeletal muscle fibrosis over time in a relatively non-invasive manner. As seen in humans with muscular dystrophy, *mdx* mice demonstrate individual variation in fibrosis. This may explain why it was the *change* in R1 value from pre-treatment to post-treatment which demonstrated a significant difference between the treated and untreated groups rather than the absolute R1 change in the post-treatment scan alone (34, 35). This is important for future experiments using EP3533, with baseline and post treatment scans being the correct way to demonstrate change, rather than a single measurement.

Other quantitative MRI methods have been used clinically in muscular dystrophies, including native T1, T2, magnetic resonance spectroscopy, and Dixon fat fraction (FF) (36–41). In particular, FF has been shown to be a sensitive measure of disease progression. A drawback of the *mdx* mouse model is that it demonstrates minimal intramuscular fat replacement compared to humans with the condition. Therefore, the utility of EP3533-enhanced MRI scan would have to be compared against FF as an outcome measure. In human pathology, fat replacement eventually becomes the dominant pathology with large percentages of muscle replaced, therefore it is unknown whether EP3533-enhanced MRI is likely to be helpful in advanced disease or to be merely used alongside established biomarkers such as FF.

In murine experiments several standardised assessments have been developed to measure motor function and strength in a reliable way (25, 34, 42). External validity may be reduced due to different compensatory mechanisms employed by both species (43). Grip strength tests in rodents are most akin to quantitative muscle assessment in humans (44), with such grip assessments able to demonstrate significant differences between control and *mdx* mice at an early age (34). In this experiment functional results correlated significantly to R1 change (*r* = −0.33, *p* = 0.004), and demonstrated a significant difference between untreated and treated *mdx* groups (*p* < 0.001). In contrast to these results, Huebner et al. who performed halofuginone studies in *mdx* mice, did not report a significant difference between treated *mdx* mice and control in grip assessment (19). This difference may be due to the smaller group sizes used by Huebner et al. (19) (*n* = 5). As with functional outcome measures in humans, other factors such as cognition, stress and motivation can be confounding variables (35). In humans with DMD, quantitative MRI such as Dixon fat fraction (FF) measurement has been shown to have higher levels of correlation to the 6 min walk test (6MWT), (*r* = −0.65, *p* < 0.001), as well as other functional assessments (45–48). The correlations seen in this experiment were lower than that of FF in humans. Any future evaluation of efficacy of EP3533-induced R1 change in clinical trials should therefore be compared with functional assessments as well as MRI measures (45, 49). The relatively weak correlation between function and EP3533-induced R1 change may be due to the non-linear development of fibrosis throughout muscle, as seen in other human and murine studies (37, 50, 51).

There have been concerns around gadolinium-based contrast agents and their repeated use in humans due to potential neurotoxicity (52). Even less is known of the safety profile of peptide-based gadolinium agents which may be excreted less readily due to their complex structure. Further preclinical studies are required to provide safety data findings used to inform a future application for registration of potential human trials before this agent can be used as an outcome measure or in clinical practise.

## Conclusion

As new therapies are trialled in muscular dystrophies there is great potential for EP3533 to be used as an outcome measure, adding important, quantifiable information relatively non-invasively. EP3533 can also be used to reduce animal numbers in murine experiments, allowing accurate quantification of fibrosis over time. Our study showed that EP3533-enhanced MRI can be used in a muscular dystrophy mouse model to demonstrate disease progression over time and to effectively monitor a treatment response.

## Data Availability Statement

The raw data supporting the conclusions of this article will be made available by the authors, without undue reservation.

## Ethics Statement

The animal study was reviewed and approved by all procedures performed were in accordance with the ethical standards of directive 2010/63/EU of the European parliament and under the auspices of the terms of the animals (scientific procedures) act 1986 and project licence PB3CA650C, authorised by the Home Secretary, Home Office, United Kingdom.

## Author Contributions

All authors have contributed considerably to the design, execution of the study or to the analysis and interpretation of data, involved in drafting or critically revising the manuscript, and have read and approved its final version.

## Conflict of Interest

PC has equity in, and is a consultant to Collagen Medical LLC which holds the patent rights to EP-3533. The remaining authors declare that the research was conducted in the absence of any commercial or financial relationships that could be construed as a potential conflict of interest.
